# Editorial: Mechanisms Linking Transport and Utilization of Metabolic Fuels to the Impact of Nutrition and Exercise Upon Health

**DOI:** 10.3389/fnut.2021.803369

**Published:** 2021-11-26

**Authors:** Gregory C. Henderson, Takeshi Hashimoto, Brian A. Irving, Tanya M. Halliday

**Affiliations:** ^1^Department of Nutrition Science, Purdue University, West Lafayette, IN, United States; ^2^Faculty of Sport and Health Science, Ritsumeikan University, Shiga, Japan; ^3^Pennington Biomedical Research Center, School of Kinesiology, Louisiana State University, Baton Rouge, LA, United States; ^4^Department of Health and Kinesiology, University of Utah, Salt Lake City, UT, United States

**Keywords:** lactate, spinal cord injury, amino acid, adipose tissue, free fatty acid

For this Research Topic, we invited researchers to submit articles that described links between energy substrate metabolism and the health benefits of exercise and diet. Exercise interventions and specific dietary approaches can improve health through various mechanisms, and the focus of the Research Topic was the transport and metabolism of energy substrates. This Editorial highlights the research and review articles that were published on the topic. The goals of the Research Topic were related to the dissemination of recent discoveries and the generation of novel hypotheses, and the topics addressed spanned across metabolic aspects of each macronutrient class.

Jacobs et al. studied responses of fat oxidation in spinal cord injury patients through assessment of responses to exercise and feeding. This work addressed a population that is at high risk for metabolic disease. The authors considered that energy substrate metabolism is highly responsive to diet and exercise, and thus they characterized fat oxidation in response to each of these factors. Their data suggest that subjects with higher physical fitness could oxidize more fat during exercise. Since cardiorespiratory fitness is related to metabolic health, the authors discovered an exciting link between energy substrate metabolism and health in this understudied clinical population. Also, on the topic of fat metabolism, Axsom et al. published a review article about adipose tissue biology. Adipose tissue lipolysis is an important regulator of lipid metabolism and fat oxidation (1). The authors reviewed the literature about epigenetic regulation of metabolism in adipose tissue and the liver, speculating how this regulation may be harnessed in the response to chronic exercise training. The authors also addressed various aspects of lipid and glucose metabolism and presented connections between control of fuel oxidation and epigenetic modifications. One intriguing suggestion by the authors was that the elevated fat oxidation occurring from free fatty acid (FFA) utilization during (or after) exercise (2, 3) might lead to enhanced acetyl-CoA availability for use as a substrate for histone acetylation. Future work could lead to a greater understanding of the implications of elevated plasma FFA abundance and utilization during and after exercise (2, 4) and in negative energy balance (5).

In the Research Topic, protein metabolism was also addressed in a study by Serrano et al. The data revealed that plasma amino acid concentrations were lower after compared to before an acute exercise bout in people who were non-obese. This decline was not apparent in people who were obese. Furthermore, neither group exhibited an increased protein synthesis rate in muscle after exercise in the fasted state. This study, conducted in the fasted state, draws attention to the important synergies between diet and exercise for controlling metabolism and health. Plasma amino acids rise in the postprandial state (6), and the authors would likely have observed enhanced synthesis rates of mitochondrial and total protein if nutrition had been provided (7, 8). Nonetheless, the findings about the fasted state lead to an intriguing observation about possible differences between lean and obese people for regulation of plasma amino acid concentrations in the fasted state, and future work could further indicate the implications of this finding.

Carbohydrate metabolism was also covered in the Research Topic. The focus in these papers was upon lactate, which acts as a carbohydrate metabolite that is readily exchanged between tissues and cellular compartments (9, 10). Considering blood glucose alone is insufficient to understand carbohydrate metabolism response to exercise and diet, as blood lactate is also a rapidly transported metabolite. Messonnier et al. studied a unique type of exercise bout, which led to new insights about metabolite concentration responses to physical stress. Following a brief yet intense exercise challenge, the authors observed that blood lactate rose transiently, followed by a rise in blood glucose levels. Furthermore, the participants with a higher lactate response to exercise showed a more pronounce post-exercise increase in blood glucose. Clearly, when considering the regulation of blood glucose in the context of health and stress physiology, as previously discussed in-depth (9, 10), lactate should be regarded as an important substrate through which carbohydrate energy is shuttled between tissues. In his review article, Brooks further addressed lactate metabolism. Brooks wrote an insightful review article about the role of the heart in systemic lactate metabolism, combining a review of concepts with new hypothesis generation. As covered in this article by the investigator who initially proposed the Cell-Cell lactate shuttle concept, Brooks describes how tissues share carbohydrate energy by transporting lactate between lactate producing and lactate consuming tissues, and the Intracellular Lactate Shuttle concept addresses lactate utilization as an oxidative fuel in mitochondria. As described in the review article, blood lactate from skeletal muscle (and other organs) during exercise is taken up by the heart and oxidized as a mitochondrial fuel. This is an example of a Cell-Cell lactate shuttle and is likely important for cardiac performance and health. Additionally, the author proposed a type of intracellular lactate shuttle by which glycolytic lactate production and mitochondrial lactate oxidation are potentially controlled to coincide with contraction cycling; surges of glycolytic lactate production may occur during cardiomyocyte contraction with mitochondrial oxidation of lactate occurring during relaxation (diastole). This hypothesis could have exciting implications for the role of carbohydrate metabolism in cardiac performance.

The transport and utilization of energy metabolites is dysfunctional in states of metabolic disease, and a greater understanding of the mechanisms linking transport and utilization of metabolic fuels to health has been needed. The handling of macronutrients within heart, skeletal muscle, liver, and adipose tissue has serious implications for regulation of metabolic health, and the articles in this Research Topic have provided new insights into this research area. From translational and clinical research, knowledge of exercise and diet prescription to achieve specific health outcomes is reasonably strong. Still, new ideas are needed to link health outcomes to the transport and metabolism of metabolic fuels. Through this Research Topic, the authors have advanced our understanding. It is clear that energy metabolism is an important factor in human health. Thus, scientific investigation into metabolism and transport of carbohydrate metabolites, lipids, and amino acids is critical for shedding light upon mechanisms through which exercise and diet affect health.

## Author Contributions

GH, THas, BI, and THal wrote the Editorial. All authors contributed to the article and approved the submitted version.

## Conflict of Interest

The authors declare that the research was conducted in the absence of any commercial or financial relationships that could be construed as a potential conflict of interest.

## Publisher's Note

All claims expressed in this article are solely those of the authors and do not necessarily represent those of their affiliated organizations, or those of the publisher, the editors and the reviewers. Any product that may be evaluated in this article, or claim that may be made by its manufacturer, is not guaranteed or endorsed by the publisher.
